# The Hidden Hernia: A Rare Sciatic Hernia Masquerading as Gluteal Pain in an Elderly Woman

**DOI:** 10.7759/cureus.92245

**Published:** 2025-09-13

**Authors:** Hosam Alazazzi, Georges Ziade, Bawan Ali, Aemon Fatima Jaffery, Huw Shopland, Martin T Antony

**Affiliations:** 1 General Surgery, Wrightington, Wigan and Leigh NHS Foundation Trust, Wigan, GBR; 2 Accident and Emergency Medicine, Wrightington, Wigan and Leigh NHS Foundation Trust, Wigan, GBR; 3 Medicine and Surgery, Royal Albert Edward Infirmary, Wigan, GBR

**Keywords:** gluteal hernia, pelvic floor hernia, rare hernia, sciatic foramen, sciatic hernia

## Abstract

Sciatic hernias are one of the rarest pelvic floor hernias and a highly uncommon cause of gluteal or abdominal pain. Their variable presentation often delays diagnosis, particularly in frail patients.

We report a case of a 68-year-old woman with severe chronic obstructive pulmonary disease and chronic comorbidities who presented with vague abdominal and gluteal pain. CT imaging revealed a left-sided sciatic hernia involving the sigmoid colon without obstruction. She underwent a successful open hernia reduction and sigmoidopexy, avoiding mesh due to high operative risk.

Sciatic hernias can mimic common conditions like diverticulitis or sciatica. Imaging is essential for diagnosis, especially in high-risk patients. In this case, a tailored surgical approach minimized operative time and complications.

This case highlights the importance of considering sciatic hernias in elderly patients with nonspecific pelvic or gluteal symptoms. Early imaging and individualized management are key to optimizing outcomes.

## Introduction

A sciatic hernia is a protrusion of intra-abdominal contents through the greater or lesser sciatic foramen, pathways normally traversed by the piriformis muscle and sciatic nerve [1]. Pelvic hernias, including obturator, sciatic, and perineal types, are exceptionally rare, accounting for less than 1% of all abdominal wall hernias [2]. Historically, these hernias were poorly understood and often diagnosed only during surgery or at autopsy. Sciatic hernias, first described in the 18th century, remain the rarest of these, with just over a hundred reported cases in the literature [1].

Sciatic hernias tend to occur more frequently in women, and several risk factors have been proposed, including significant weight loss, multiparity, and intra-abdominal masses such as liposarcomas [2,3]. In some cases, they coexist with other hernia types [4]. The underlying pathophysiology is often unclear but is thought to involve elevated intra-abdominal pressure and atrophy of the piriformis muscle, which forms part of the boundary of the greater sciatic foramen [2,3]. Because of its anatomical proximity to the sciatic nerve, sciatic hernias may mimic radicular symptoms, such as buttock or lower limb pain, which can complicate and delay diagnosis [2].

In the UK, these hernias are so uncommon that national data are sparse, with only a few dozen cases likely treated surgically each year [5]. While their overall healthcare burden is small compared to more common hernias, pelvic hernias carry a disproportionately high risk of complications, and delayed diagnosis may result in significant morbidity or mortality. Early imaging and timely surgical intervention are therefore critical in improving patient outcomes [2,3].

This case highlights a rare instance of a sciatic hernia identified incidentally during physical examination and imaging in a frail elderly patient presenting with vague abdominal and gluteal symptoms. The absence of bowel obstruction and the patient’s high operative risk added complexity to both diagnosis and management. Given the scarcity of reported cases and the unique anatomical and clinical considerations involved, this report contributes to the limited literature on sciatic hernias and offers insight into a tailored surgical approach in a high-risk setting.

## Case presentation

A 68-year-old woman presented with a five-day history of intermittent left-sided abdominal pain, abdominal distension, and reduced bowel movements, passing only small, hard stools despite regular use of macrogol and lactulose. She reported one episode of bilious vomiting but denied blood or mucus in the stool, recent surgeries, fevers, or malaise. 

Her medical history included severe chronic obstructive pulmonary disease (COPD), osteoporosis, hypertension, right-sided lung cancer, and colonic diverticulosis. She had no known drug allergies. She was a former smoker with a surgical history of open cholecystectomy and laparoscopic bilateral tubal ligation. Functionally, she was frail, breathless at rest, led a sedentary lifestyle, required assistance with daily activities, and used a walking stick indoors. 

On examination, she was hemodynamically stable. Her abdomen was soft with mild tenderness in the left iliac fossa, without peritonism or palpable inguinal hernias. Moderate tenderness was noted in the left mid-gluteal region with a tender, slipping mass that was small, not reducible, and showed no cough impulse. Its size was difficult to assess accurately due to significant discomfort and the deep anatomical location. Digital rectal and neurological examinations were normal, with no signs of sciatica. Laboratory tests, including full blood count, inflammatory markers, renal function, lactate, and urinalysis, were unremarkable as shown in Table 1.

**Table 1:** Blood test result summary. WBC: White Blood Cell; INR: International Normalized Ratio

Blood test	Value	Unit	Reference range
Hemoglobin	111	g/L	130-180
Neutrophils	2.9	x10^9/L	1.8-7.5
WBC	7.8	x10^9/L	4.0-11.0
Lymphocytes	1.8	x10^9/L	1.0-4.0
Platelets	277	x10^9/L	150-450
Sodium	138	µmol/L	133-146
Potassium	4.2	µmol/L	3.5-5.3
Creatinine	47	µmol/L	47-104
Urea	3.4	µmol/L	2.5-7.8
C-Reactive Protein	82	mg/L	<4
INR	0.9	U/L	0.8-1.2

Given this constellation of abdominal and gluteal findings in a frail elderly patient, the differential diagnosis included diverticulitis, constipation-related pain, gluteal abscess, obturator hernia, and sciatica.

**Figure 1:**
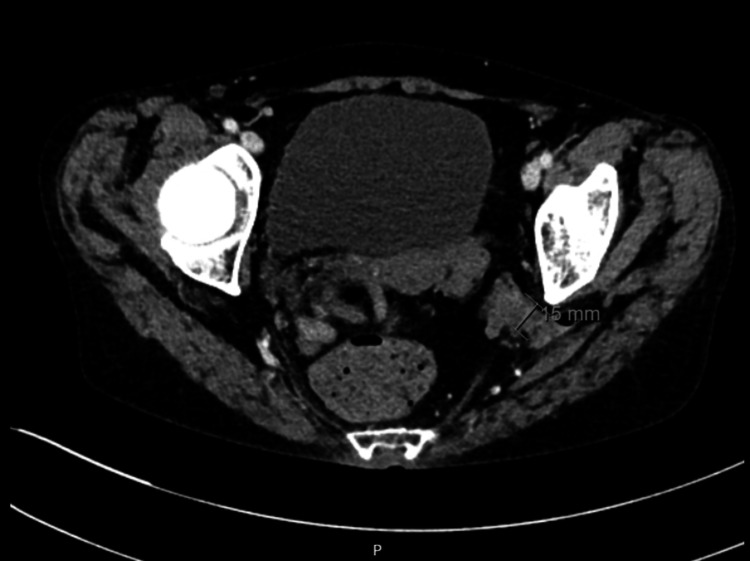
Axial CT image showing a left-sided sciatic hernia measuring approximately 15 mm, protruding through the greater sciatic foramen.

**Figure 2:**
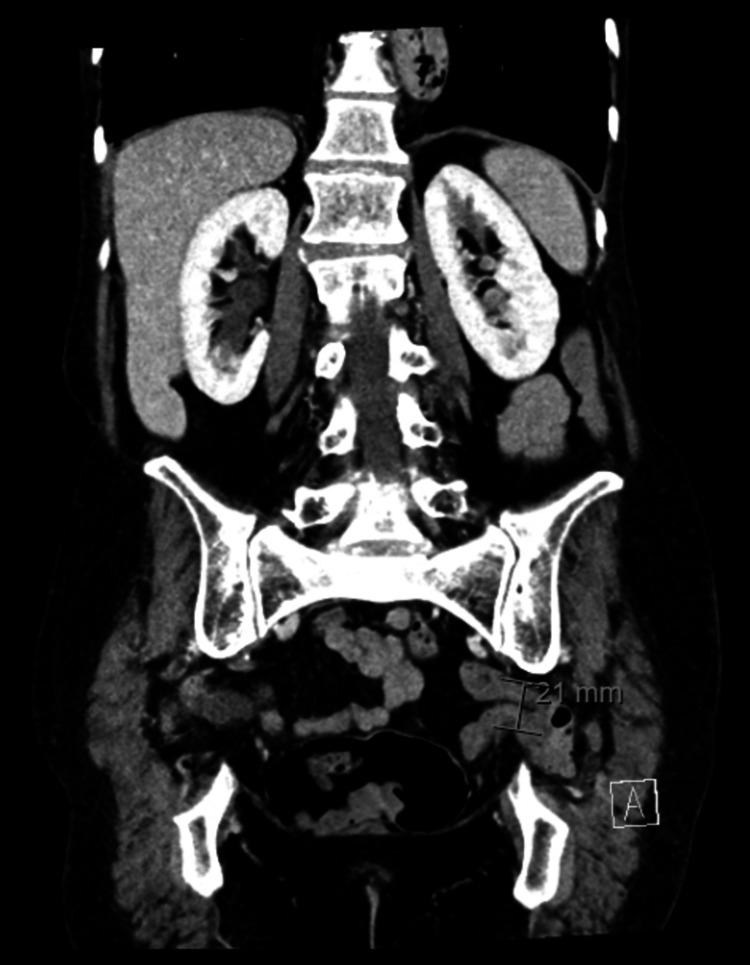
Coronal CT image demonstrating the same left-sided sciatic hernia with a maximum dimension of 21 mm.

A contrast-enhanced CT scan of the abdomen and pelvis revealed non-complicated colonic diverticulosis and an incidental left-sided sciatic hernia with large bowel loops herniating through the greater sciatic foramen into the posterior infrapiriform space, without evidence of obstruction or ischemia, as seen in Figures 1, 2. The gluteal discomfort was attributed to mechanical traction or compression of the bowel within the foramen.

The patient was initially managed conservatively with nil per os (NPO) status, intravenous fluids, parenteral cyclizine 50 mg three times daily, and intravenous paracetamol 1 g four times daily. 

A multidisciplinary discussion involving surgical and anesthetic teams addressed her high operative risk due to severe breathlessness at rest, COPD, frailty, and limited physiological reserve. Shared decision-making with the patient and her daughter included counseling on risks, including postoperative pneumonia, stoma formation, ineligibility for intensive care escalation, and high perioperative mortality.

Despite these risks, the patient expressed a strong preference to proceed with surgical intervention, citing worsening discomfort and declining quality of life. She consented to surgery with full understanding of the potential outcomes and confirmed she did not wish to be resuscitated in the event of cardiac arrest.

Intraoperative findings

A Pfannenstiel incision was performed. A multidisciplinary discussion in the operating room with the surgical and anesthetic teams addressed her high operative risk and guided the choice of approach. A redundant sigmoid colon was found herniating through the sciatic foramen, with no obstruction, strangulation, or ischemia. The hernia was reduced, and sigmoidopexy was performed to anchor the colon to the pelvic brim in order to prevent recurrence. Mesh placement and deep dissection were avoided to minimize operative time and complications in this high-risk patient. The procedure was uncomplicated, with minimal blood loss and no need for bowel resection.

Post-operative findings

Postoperatively, the patient received analgesia, intravenous fluids, and gradual reintroduction of oral intake. Close monitoring for respiratory complications, early mobilization, and chest physiotherapy were prioritized. She recovered without immediate complications. Three weeks post-surgery, she presented to the emergency department with nausea and vomiting. A CT scan showed no acute abnormalities, and she was discharged after 16 hours of observation.

## Discussion

This case contributes to the limited literature on sciatic hernias, a rare pelvic floor condition that poses significant diagnostic and therapeutic challenges due to its infrequent occurrence and vague clinical presentation [2]. Sciatic hernias involve the protrusion of peritoneal contents through the greater or lesser sciatic foramen, with fewer than 100 cases documented worldwide [1,2]. They are categorized into three subtypes based on their anatomical location: suprapiriform, which accounts for 60% of cases and involves protrusion above the piriformis muscle alongside the superior gluteal artery and nerve; infrapiriform, comprising 30% of cases, where the hernia follows the path of the inferior gluteal vessels, internal pudendal vessels, and sciatic nerve; and subspinous, representing 10% of cases, where the hernia exits through the lesser sciatic foramen, positioned medially to the internal pudendal vessels and sciatic nerve [2,6].

The ambiguous clinical features of sciatic hernias complicate their detection. Patients may experience nonspecific abdominopelvic discomfort, bowel obstruction, gluteal abscesses, or, rarely, a reducible mass near the sciatic notch [1,3,4]. Small hernias are often obscured by the gluteus maximus muscle, especially in obese individuals, and sciatica-like neurological symptoms arise only when the sciatic nerve is compressed [5]. In this case, a contrast-enhanced CT scan incidentally revealed a sigmoid colon loop within the greater sciatic foramen, highlighting the essential role of advanced imaging in evaluating unclear pelvic or gluteal symptoms in vulnerable groups, such as elderly or frail patients with multiple comorbidities [2,6,7]. 

Computed tomography (CT) is the preferred imaging modality for precisely identifying sciatic hernias and distinguishing them from other conditions, such as obturator hernias or gluteal abscesses. Ultrasonography, initially documented for hernia diagnosis in 1975, can detect the hernia sac as a hypoechoic tubular structure, with color Doppler enabling evaluation of blood flow and bowel viability [4,7]. However, the operator-dependent nature of ultrasonography reduces its reliability for detecting deep pelvic hernias [7]. Although not utilized in this case, MRI provides excellent soft-tissue contrast, making it valuable for assessing pelvic floor integrity and nerve involvement [8].

The surgical management of sciatic hernias requires individualization, particularly in medically complex patients, as no standardized operative approaches exist [3,4]. Various laparoscopic and open (laparotomy) techniques have been reported, with the surgical approach often tailored based on hernia location, surgeon experience, and patient factors [9-11]. In our case, a laparotomy was selected due to the surgical team’s familiarity with this rare hernia type, the hernia’s infrapiriform position requiring direct visualization, and the patient’s smaller body habitus. This approach offered superior visual and tactile feedback, as well as reduced operative time during the sigmoidopexy. Conversely, laparoscopic and robotic-assisted repairs with mesh have been associated with shorter recovery times, fewer complications, and smaller incisions in other reports [10,11]. However, no large-scale comparative studies currently exist to define optimal operative strategies for sciatic hernias.

The patient’s severe COPD, frailty, and functional dependence posed significant perioperative risks, necessitating a tailored approach. While transabdominal or transgluteal repairs with mesh placement are commonly described, these may be unsuitable for high-risk patients due to prolonged operative time or increased complication rates [2,4]. Sigmoidopexy, performed in this case to reduce the herniated sigmoid colon and prevent recurrence, represents a less invasive alternative that balances efficacy and operative safety, avoiding the risks of mesh placement in a frail patient [4]. Although infrapiriform hernias often course alongside the sciatic nerve, no clinical or radiologic evidence of nerve compression was observed in this patient [2,6,7]. However, long-term outcomes of sigmoidopexy remain understudied, highlighting the need for further research to establish optimal strategies for sciatic hernia repair [2].

This case highlights the importance of maintaining a high index of suspicion for sciatic hernias in elderly or frail patients with vague pelvic, gluteal, or abdominopelvic symptoms, particularly those with risk factors such as chronic respiratory conditions or pelvic floor weakness [1,2,6]. Shared decision-making was critical, given the patient’s high perioperative risk, emphasizing the need to align treatment with patient preferences and quality of life goals.

## Conclusions

In conclusion, we report a rare sciatic hernia in a frail elderly patient that was diagnosed via CT imaging and successfully managed with a mesh-free sigmoidopexy. This case underscores the importance of maintaining a high index of suspicion and utilizing appropriate imaging for prompt diagnosis of unusual pelvic hernias. It also demonstrates that a tailored surgical approach without mesh can be an effective option in select high-risk individuals. Given the paucity of reported sciatic hernia cases and the lack of consensus on optimal repair techniques, further case reports and studies are warranted to guide best practices and improve outcomes for this rare condition.
